# Prevalence and socio-demographic correlates of probable depression and anxiety symptoms in Mozambique: A secondary data analysis

**DOI:** 10.1371/journal.pmen.0000169

**Published:** 2025-01-30

**Authors:** Roger Antabe, Gregory Antabe, Yujiro Sano, Evans Batung

**Affiliations:** 1 Department of Health and Society, University of Toronto Scarborough, Toronto, Canada; 2 Department of Mathematics, Our Lady of Apostles School, Ahinsan Kumasi, Ashanti Region, Kumasi, Ghana; 3 Department of Sociology, Nipissing University, North Bay, Canada; 4 Department of Geography, Western University, Social Science Centre, London, Ontario, Canada; PLOS: Public Library of Science, UNITED KINGDOM OF GREAT BRITAIN AND NORTHERN IRELAND

## Abstract

There is limited research at the national level in Mozambique that examines the prevalence and correlates of depression and anxiety. Therefore our study examined the prevalence and correlates of probable depression and anxiety symptoms among women and men in the country. We used the 2022–23 Mozambique Demographic and Health Survey, specifically drawing on some socio-demographic and mental health variables measured by PHQ-9 and GAD-7 and applied multivariate logistic regression analysis. Our findings revealed high levels of depression and anxiety, that is, 10% (95% CI = 9%-12%) and 11% (95% CI = 10%-12%), respectively, for women. Among women, those from the poorer (aOR = 0.68, 95% CI = 0.48, 0.97; aOR = 0.66, 95% CI = 0.48, 0.91), middle (aOR = 0.55, 95% CI = 0.88, 0.81; aOR = 0.58, 95% CI = 0.40, 0.83), richer (aOR = 0.63, 95% CI = 0.41, 0.94; aOR = 0.63, 95% CI = 0.43, 0.91) and richest households (aOR = 0.39, 95% CI = 0.24, 0.65; aOR = 0.43, 95% CI = 0.27, 0.67) were all less likely to report symptoms of depression and anxiety, respectively. Furthermore, the employed (aOR = 0.65, 95% CI = 0.53, 0.80; aOR = 0.68, 95% CI = 0.55, 0.83) had a reduced probability of indicating they had depression and anxiety, respectively. Among men, 2% (95% CI = 2%-3%) reported depression and anxiety. While men with secondary-level educational attainment (aOR = 3.20, 95% CI = 1.19, 8.62) were more likely to report symptoms of depression, this was not the case for anxiety where those with no education (aOR = 0.13, 95% CI = 0.02, 0.78) and primary education (aOR = 0.27, 95% CI = 0.09, 0.80) were all less likely to report anxiety disorders compared to their colleagues with the highest level of educational attainment. Finally, men in rural areas (aOR = 0.40, 95% CI = 0.22, 0.74) relative to their urban counterparts were less likely to report depression although this was not significant for anxiety disorders. Mental health policy in Mozambique needs to pay critical attention to groups such as older age cohorts, the formerly married, Muslim women and people with lower socioeconomic status.

## Introduction

Mental health illnesses, such as probable depression and anxiety in low-income settings in Sub-Saharan Africa (SSA), including Mozambique, are emerging as major public health concerns. The World Health Organization (WHO) estimates that 332 and 264 million people globally are living with depression and anxiety, respectively, translating into 4.4% and 3.6% prevalence rates. In SSA, however, the prevalence rates for depression and anxiety rise to as much as 9% and 10%, respectively. Beyond these notable increases, an even more worrisome reality may exist. First, these rates are rather conservative due to under-reporting [1]. For example, Jorns-Presentati et al. (2021) [2] established that depression was as high as 26.9% among adolescents in SSA with the authors observing a similar trend in the general population. Another stream of complexity is the multi-faceted burden of disease. The high prevalence rates may have further worsened during COVID-19, where across the African region, the prevalence of depression and anxiety were reported at 48% and 47%, respectively [3].

These trends are reflective of the context of Mozambique, where mental health conditions such as schizophrenia, probable depression and anxiety disorders pose a significant threat to the country’s public health system. These have resulted in calls for increased governmental intervention [4]. Citing the 2019 Ministry of Health Mozambique Mental Health Annual Report, Muanido et al. [5] observed that 6% of reported cases of mental health in hospitals were for mood disorders, inclusive of depression, while another 5% was for neurotic, stress-related and somatoform disorders that included anxiety disorders. Given that these estimates were those seeking mental healthcare in formal healthcare facilities, it is argued that the prevalence of probable depression and anxiety disorders among the general Mozambican population would be higher. In the Sofala and Manica Provinces of the country for instance, recent estimates suggest that while 19% of people reported depressive symptoms and another 17% indicating lifetime suicide ideation, only 10% of the general population and 26% of those reporting mental illness ever sought care for their health condition [6]. In rural districts in Central Mozambique, Audet et al. [4] also found depression to be as high as 17% among female-headed households.

In response to these calls, the Mozambican government has strategized to reduce the increasing incidence of mental illnesses through action areas outlined in the *Programa de saúde mental 2006–2015* [7]. Despite these efforts, the rising trends has been partly sustained by high levels of misconceptions about mental illnesses in the country [8]. For example, many Mozambicans perceive mental illness as a “spiritual condition”, and thus requiring primary attention from spiritual and traditional health practitioners. This observation in Mozambique is particularly concerning as the high prevalence rates of mental health conditions such as probable depression and anxiety may also underlie other public health challenges in the country, including the high rates of suicide—ranked as the sixth highest globally. While these dynamics call for an in-depth understanding of the factors associated with probable depression and anxiety symptoms to inform a holistic public health policy, there have been nascent studies on mental health in the country. To contribute to the literature and inform mental health policy in Mozambique, the key objective of our study is to understand the prevalence and socio-demographic correlates of probable depression and anxiety symptoms among women and men in Mozambique.

To understand the prevalence and correlates of probable depression and anxiety symptoms in Mozambique, we glean insights from earlier scholars who have noted several factors that may be associated with depression and anxiety disorders. These have broadly been classified as demographic, socioeconomic and locational characteristics of people. Considering the demographic aspect, scholars have alluded to the fact that differences in hormones, the socialization process and life stress may contribute to more women reporting depression and anxiety symptoms relative to their male counterparts [1,9]. Similarly, older age and the ever-married—those divorced, separated or widowed—tend to have a higher prevalence of depression and anxiety relative to their younger and never-married counterparts [6,10,11]. Additionally, those who hold religious beliefs have been found more likely to have depression and anxiety compared to those without religious beliefs [12,13]. The role of socioeconomic factors on depression and anxiety has also been clearly acknowledged in the literature. That is, people with low income, low or no levels of educational attainment and the unemployed persistently indicate a higher prevalence of depression and anxiety symptoms [14–16]. Finally, it has been established that urban residents are better positioned to be resilient against mental illnesses, leading to more people in rural areas reporting depression and anxiety compared to their urban colleagues [17].

## Methods

The study utilized secondary data from the Mozambique Demographic and Health Survey (MDHS) conducted from July 2022 to March 2023. The MDHS is a nationwide survey that is collected by the Institute National Statistics, employing standardized questionnaires and a multi-stage stratified cluster sampling method. This method included stratification by province and urban/rural areas, with equal probability sampling for primary sampling units and proportional sampling for enumeration areas. The MDHS provides high quality and reliable information on basic demographic indices and mental health such as PHQ-9 and GAD-7. The interviews were completed with 13,183 women aged 15–49 and 5,380 men aged 15–54 with a response rate of 94% and 86% for women and men, respectively.

### Ethics statement

The survey protocol, such as the biological measurement and test procedures, was appraised and approved by the Instituto Nacional de Estatística Maputo, Moçambique and the Institutional Review Board of ICF, Rockville, Maryland, USA. Interviewers obtained informed consent by reading the consent statement of the respondents, who may accept or decline to participate.

### Measures

In our analysis, we utilized the PHQ-9 and GAD-7 as dependent variables to assess mental health symptoms related to depression and anxiety. The PHQ-9 is a widely used self-report scale to measure the severity of depression, consisting of 9 items rated on a 4-point scale, with strong psychometric properties with robust internal consistency (α = 0.90 and α = 0.80 for women and men, respectively). For the PHQ-9, scores were categorized into "depressed" (scores of 10 or above, indicating moderate to severe depression) and "not depressed" (scores below 10). Similarly, the GAD-7 measures the severity of generalized anxiety disorder symptoms with 7 items rated on a scale from 0 to 3, demonstrating robust internal consistency (α = 0.91 and α = 0.78 *for women and men*, *respectively*). To streamline the data analysis and enhance interpretability, we created a binary variable derived from these scores. Specifically, for the PHQ-9 scores, were categorized into "depressed" (indicating moderate to severe depression with scores of 10 or above) and "not depressed" (scores below 10). Similarly, the GAD-7 scores were classified into "anxious" (indicating moderate to severe anxiety with scores of 10 or above) and "not anxious" (scores below 10). This binary classification, based on clinical guidelines, clearly distinguishes between people experiencing significant symptoms and those who are not, enabling a more focused analysis of the associations and impacts of depression and anxiety in our study population [18,19]. For independent variables, we included socioeconomic and demographic variables. For socioeconomic variables, we considered education (0 = no education; 1 = primary education; 2 = secondary education; 4 = higher education), household wealth (0 = poorest; 1 = poorer; 2 = middle; 3 = richer; 4 = richest), and employment status (0 = unemployed; 1 = employed). We also added four demographic variables, such as age (0 = 15–19; 1 = 20–24; 2 = 25–29; 3 = 30–34; 4 = 35–39; 5 = 40–44; 6 = 45–49; 7 = 50–54), place of residence (0 = urban; 1 = rural), religion (0 = Muslim; 1 = Catholic; 2 = Zion Christian; 3 = Evangelical/Pentecostal; 4 = no religion), and marital status (0 = formerly married; 1 = never married; 2 = currently married).

### Analysis

We conducted three distinct analyses to examine our data. First, descriptive statistics were employed to outline the characteristics of our analytical sample. Next, bivariate analysis was used to explore the unadjusted relationships between the dependent and independent variables. To account for socioeconomic and demographic factors simultaneously, we performed multivariate analysis. Given the dichotomous nature of our dependent variables, logit models were utilized for both bivariate and multivariate analyses. All analyses were carried out using STATA 17 (StataCorp, College Station, TX, USA), with the ‘svy’ function applied to account for the cluster sampling design and sampling weights.

## Results

Table 1 provides an overview of the sample characteristics for women (n = 13,183) and men (n = 5,380) in Mozambique. Among women, 10% (95% CI = 9%-12%) had PHQ-9 scores of 10 or higher, indicating significant depressive symptoms, compared to just 2% (95% CI = 2%-3%) of men. For anxiety, as measured by the GAD-7, 11% (95% CI = 10%-12%) of women scored 10 or higher, while only 2% (95% CI = 2%-3%) of men did. Educational attainment varied between genders, with 27% (95% CI = 25%-28%) of women having no formal education compared to 11% of men, and a higher proportion of women (28%; 95% CI = 26%-30%) having secondary education versus men (38%; 95% CI = 35%-40%). Employment status showed a notable gender difference, with 70% (95% CI = 68%-71%) of women being unemployed versus 18% (95% CI = 17%-21%) of men. Age distribution was similar across genders, with most individuals residing in rural areas (61% of women and 60% of men). Marital status also showed minor differences between the two groups, with more women currently married (64%; 95% CI = 63%-66%) compared to men (58%; 95% CI = 56%-60%).

**Table 1:** Sample characteristics of women (n = 13,183) and men (n = 5,380) in Mozambique.

	*Women*	*Men*
	*Percentage (95% CI)*	*Percentage (95% CI)*
*PHQ-9*		
Less than 10	90 (88, 91)	98 (97, 98)
10 or higher	10 (9, 12)	2 (2, 3)
*GAD-7*		
Less than 10	89 (88, 90)	98 (97, 98)
10 or higher	11 (10, 12)	2 (2, 3)
*Education*		
No education	27 (25, 28)	11 (9, 13)
Primary education	42 (41, 44)	47 (45, 50)
Secondary education	28 (26, 30)	38 (35, 40)
Higher education	3 (2, 3)	4 (3, 5)
*Household wealth*		
Poorest	18 (17, 20)	16 (14, 18)
Poorer	18 (16, 19)	19 (17, 21)
Middle	18 (17, 20)	18 (16, 20)
Richer	21 (20, 23)	20 (17, 22)
Richest	25 (23, 26)	27 (25, 30)
*Employment status*		
Unemployed	70 (68, 71)	18 (17, 21)
Employed	30 (29, 32)	82 (79, 83)
*Age*		
15–19	23 (22, 24)	26 (24, 27)
20–24	20 (19, 21)	18 (17, 20)
25–29	17 (16, 18)	15 (13, 16)
30–34	12 (11, 13)	12 (11, 13)
35–39	11 (11, 12)	9 (8, 10)
40–44	9 (8, 10)	8 (7, 9)
45–49	8 (7, 8)	7 (6, 8)
50–54	NA	5 (4, 6)
*Place of residence*		
Urban	39 (37, 41)	40 (38, 43)
Rural	61 (59, 63)	60 (57, 62)
*Religion*		
Muslim	21 (18, 23)	21 (18, 24)
Catholic	30 (27, 32)	29 (26, 32)
Zion Christian	12 (11, 13)	9 (8, 11)
Evangelical/Pentecostal	28 (27, 30)	26 (24, 28)
No religion	7 (6, 8)	12 (11, 14)
Other	2 (2, 3)	3 (2, 3)
*Marital status*		
Formerly married	14 (13, 14)	5 (4, 6)
Never married	22 (21, 23)	37 (35, 39)
Currently married	64 (63, 66)	58 (56, 60)
*Total*	100	100

As shown in Table 2, the unadjusted logit models reveal that women with no education (OR = 2.35, 95% CI = 1.39, 3.95) and primary education (OR = 2.40, 95% CI = 1.44, 3.99) had higher odds of having PHQ-9 scores of 10 or higher compared to those with higher education. Similarly, women in the poorer (OR = 0.64, 95% CI = 0.46, 0.91), middle (OR = 0.50, 95% CI = 0.35, 0.72), richer (OR = 0.64, 95% CI = 0.44, 0.92), and richest (OR = 0.34, 95% CI = 0.24, 0.48) wealth categories had lower odds of having PHQ-9 scores of 10 or higher compared to those in the poorest wealth category. Employed women had lower odds (OR = 0.52, 95% CI = 0.42, 0.65) of having PHQ-9 scores of 10 or higher compared to unemployed women. For GAD-7 scores of 10 or higher, women with no education (OR = 1.95, 95% CI = 1.22, 3.12) and primary education (OR = 2.00, 95% CI = 1.28, 3.12) had higher odds compared to those with higher education. Women in the poorer (OR = 0.62, 95% CI = 0.46, 0.84), middle (OR = 0.53, 95% CI = 0.38, 0.75), richer (OR = 0.66, 95% CI = 0.47, 0.97), and richest (OR = 0.40, 95% CI = 0.30, 0.54) wealth categories had lower odds of GAD-7 scores of 10 or higher compared to the poorest wealth category, while employed women had reduced odds (OR = 0.59, 95% CI = 0.48, 0.72) compared to unemployed women. Regarding age, women aged 20–24 (OR = 1.33, 95% CI = 1.04, 1.71) and 35–39 (OR = 1.34, 95% CI = 1.03, 1.76) had higher odds of having PHQ-9 scores of 10 or higher compared to those aged 15–19. Similarly, Women aged 20–24 (OR = 1.41, 95% CI = 1.10, 1.81), 25–29 (OR = 1.53, 95% CI = 1.18, 1.99), 30–34 (OR = 1.36, 95% CI = 1.02, 1.82), 35–39 (OR = 1.81, 95% CI = 1.40, 2.35) and 45–49 (OR = 1.55, 95% CI = 1.14, 2.12) also had higher odds of having GAD-7 scores of 10 or higher compared to younger age groups. Women identifying as Catholic (OR = 0.70, 95% CI = 0.52, 0.93), Zion Christian (OR = 0.24, 95% CI = 0.16, 0.34), Evangelical/Pentecostal (OR = 0.35, 95% CI = 0.25, 0.49), or with no religion (OR = 0.52, 95% CI = 0.35, 0.78) had lower odds of having GAD-7 scores of 10 or higher compared to Muslims. Similarly, these groups had lower odds of having PHQ-9 scores of 10 or higher, with Catholics (OR = 0.72, 95% CI = 0.53, 0.99), Zion Christians (OR = 0.24, 95% CI = 0.16, 0.37), Evangelical/Pentecostals (OR = 0.36, 95% CI = 0.26, 0.49), and those with no religion (OR = 0.48, 95% CI = 0.31, 0.73) showing reductions in depressive symptoms compared to Muslims. Women who were never married had lower odds (OR = 0.64, 95% CI = 0.48, 0.87) of having PHQ-9 scores of 10 or higher and those who were currently married had reduced odds (OR = 0.76, 95% CI = 0.61, 0.96) of having GAD-7 scores of 10 or higher compared to those who were formerly married.

**Table 2:** Unadjusted and adjusted logit models predicting PHQ-9 and GAD-7 among women in Mozambique.

	Unadjusted models						Adjusted models					
	*PHQ-9*			*GAD-7*			*PHQ-9*			*GAD-7*		
	*OR*	*95% CI*		*OR*	*95% CI*		*OR*	*95% CI*		*OR*	*95% CI*	
*Education*												
No education	2.35	1.39	3.95	1.95	1.22	3.12	1.22	0.68	2.21	1.00	0.60	1.68
Primary education	2.40	1.44	3.99	2.00	1.28	3.12	1.50	0.85	2.66	1.29	0.80	2.09
Secondary education	1.48	0.89	2.48	1.47	0.96	2.25	1.18	0.69	2.03	1.28	0.83	1.97
Higher education (reference)	1.00			1.00			1.00			1.00		
*Household wealth*												
Poorest (reference)	1.00			1.00			1.00			1.00		
Poorer	0.64	0.46	0.91	0.62	0.46	0.84	0.68	0.48	0.97	0.66	0.48	0.91
Middle	0.50	0.35	0.72	0.53	0.38	0.75	0.55	0.38	0.81	0.58	0.40	0.83
Richer	0.64	0.44	0.92	0.66	0.47	0.93	0.62	0.41	0.94	0.63	0.43	0.91
Richest	0.34	0.24	0.48	0.40	0.30	0.54	0.39	0.24	0.65	0.43	0.27	0.67
*Employment status*												
Unemployed (reference)	1.00			1.00			1.00			1.00		
Employed	0.52	0.42	0.65	0.59	0.48	0.72	0.65	0.53	0.80	0.68	0.55	0.83
*Age*												
15–19 (reference)	1.00			1.00			1.00			1.00		
20–24	1.33	1.04	1.71	1.41	1.10	1.81	1.30	0.98	1.73	1.27	0.97	1.65
25–29	1.30	1.00	1.69	1.53	1.18	1.99	1.32	0.96	1.82	1.40	1.01	1.94
30–34	1.14	0.86	1.51	1.36	1.02	1.82	1.25	0.89	1.75	1.35	0.94	1.94
35–39	1.34	1.03	1.76	1.81	1.40	2.35	1.50	1.10	2.06	1.86	1.35	2.58
40–44	0.94	0.71	1.23	1.11	0.81	1.51	1.05	0.72	1.55	1.17	0.80	1.71
45–49	1.36	0.99	1.85	1.55	1.14	2.12	1.46	1.01	2.10	1.57	1.09	2.24
*Place of residence*												
Urban (reference)	1.00			1.00			1.00			1.00		
Rural	1.12	0.83	1.50	1.04	0.80	1.37	0.71	0.49	1.02	0.73	0.52	1.03
*Religion*												
Muslim (reference)	1.00			1.00			1.00			1.00		
Catholic	0.72	0.53	0.99	0.70	0.52	0.93	0.74	0.55	0.99	0.70	0.53	0.92
Zion Christian	0.24	0.16	0.37	0.24	0.16	0.34	0.29	0.19	0.44	0.27	0.18	0.39
Evangelical/Pentecostal	0.36	0.26	0.49	0.35	0.25	0.49	0.46	0.34	0.63	0.42	0.30	0.58
No religion	0.48	0.31	0.73	0.52	0.35	0.78	0.52	0.34	0.79	0.56	0.37	0.85
Other	0.27	0.14	0.53	0.38	0.20	0.71	0.31	0.16	0.60	0.42	0.22	0.77
*Marital status*												
Formerly married (reference)	1.00			1.00			1.00			1.00		
Never married	0.64	0.48	0.87	0.51	0.38	0.67	0.79	0.52	1.18	0.62	0.43	0.89
Currently married	0.79	0.63	1.00	0.76	0.61	0.96	0.73	0.57	0.93	0.72	0.57	0.91

In the adjusted models, as also shown in Table 2, higher wealth was associated with lower odds of both conditions, with women in poorer (aOR = 0.68, 95% CI = 0.48, 0.97), middle (aOR = 0.55, 95% CI = 0.38, 0.81), richer (aOR = 0.62, 95% CI = 0.41, 0.94), and richest (aOR = 0.39, 95% CI = 0.24, 0.65) households showing progressively reduced odds of having PHQ-9 scores of 10 or higher. Similarly, higher wealth was associated with lower odds of both conditions, with women in poorer (aOR = 0.66, 95% CI = 0.48, 0.91), middle (aOR = 0.58, 95% CI = 0.40, 0.83), richer (aOR = 0.63, 95% CI = 0.43, 0.91), and richest (aOR = 0.43, 95% CI = 0.27, 0.67) households showing progressively reduced odds of having GAD-7 scores of 10 or higher. Employed women had lower odds of both PHQ-9 scores of 10 or higher (aOR = 0.65, 95% CI = 0.53, 0.80) and GAD-7 scores of 10 or higher (aOR = 0.68, 95% CI = 0.55, 0.83) compared to unemployed women. Age was positively associated with both conditions, with higher odds observed for women aged 35–39 (aOR = 1.50, 95% CI = 1.10, 2.06 for PHQ-9; aOR = 1.86, 95% CI = 1.35, 2.58 for GAD-7) and 45–49 (aOR = 1.46, 95% CI = 1.01, 2.10 for PHQ-9; aOR = 1.57, 95% CI = 1.09, 2.24 for GAD-7). In terms of religion, women identifying as Catholic (aOR = 0.74, 95% CI = 0.55, 0.99), Zion Christian (aOR = 0.29, 95% CI = 0.19, 0.44), Evangelical/Pentecostal (aOR = 0.46, 95% CI = 0.34, 0.63), or with no religion (aOR = 0.52, 95% CI = 0.34, 0.79) had lower odds of having PHQ-9 scores of 10 or higher compared to Muslims. Similarly, women identifying as Catholic (aOR = 0.70, 95% CI = 0.53, 0.92), Zion Christian (aOR = 0.27, 95% CI = 0.18, 0.39), Evangelical/Pentecostal (aOR = 0.42, 95% CI = 0.30, 0.58), or with no religion (aOR = 0.556, 95% CI = 0.37, 0.85) had lower odds of having GAD-7 scores of 10 or higher compared to Muslims. In addition, women who were currently married (aOR = 0.73, 95% CI = 0.57, 0.93) had lower odds of having PHQ-9 scores of 10 or higher compared to those who were formerly married, with never married (aOR = 0.62, 95% CI = 0.43, 0.89) and currently married (aOR = 0.72, 95% CI = 0.57, 0.91) women having reduced odds of having GAD-7 scores of 10 or higher.

Results from logit models among men are shown in Table 3. In terms of unadjusted results, men with primary education had lower odds of having GAD-7 scores of 10 or higher (OR = 0.36, 95% CI = 0.15, 0.83) compared to those with higher education. Also, men from the richest household were more likely to have PHQ-9 scores of 10 or higher compared those from the poorest household (OR = 2.09, 95% CI = 1.04, 4.20). Employed men had lower odds of having PHQ-9 scores of 10 or higher (OR = 0.57, 95% CI = 0.36, 0.90). Men residing in rural areas had significantly lower odds of having PHQ-9 scores of 10 or higher (OR = 0.37, 95% CI = 0.22, 0.63) and GAD-7 scores of 10 or higher (OR = 0.56, 95% CI = 0.35, 0.89) compared to those in urban areas. Men identifying as Zion Christian (OR = 0.42, 95% CI = 0.18, 0.97) had lower odds of having GAD-7 scores of 10 or higher compared to Muslims. In addition, men who were never married had lower odds of having PHQ-9 scores of 10 or higher (OR = 0.32, 95% CI = 0.17, 0.59) and GAD-7 scores of 10 or higher (OR = 0.31, 95% CI = 0.15, 0.64). Currently married men also had lower odds of having PHQ-9 scores of 10 or higher (OR = 0.28, 95% CI = 0.15, 0.53) and GAD-7 scores of 10 or higher (OR = 0.34, 95% CI = 0.17, 0.68) compared to those who were formerly married. In the adjusted models, also shown in Table 3, men with secondary education had higher odds of having PHQ-9 scores of 10 or higher (aOR = 3.20, 95% CI = 1.19, 8.62) while those with no education (aOR = 0.13, 95% CI = 0.02, 0.78) and primary education (aOR = 0.27, 95% CI = 0.09, 0.80) had lower odds of having GAD-7 score of 10 or higher. Employed men had lower odds of having PHQ-9 scores of 10 or higher (aOR = 0.51, 95% CI = 0.30, 0.85). Men aged 35–39 (aOR = 3.39, 95% CI = 1.20, 9.55) had higher odds of having PHQ-9 scores of 10 or higher compared to those aged 15–19. Rural residence remained associated with lower odds of having PHQ-9 scores of 10 or higher (aOR = 0.40, 95% CI = 0.22, 0.74). Men who were currently married had lower odds of having both PHQ-9 scores of 10 or higher (aOR = 0.33, 95% CI = 0.18, 0.63) and GAD-7 scores of 10 or higher (aOR = 0.34, 95% CI = 0.16, 0.72) compared to those who were formerly married.

**Table 3:** Unadjusted and adjusted logit models predicting PHQ-9 and GAD-7 among men in Mozambique.

	Unadjusted models						Adjusted models					
	*PHQ-9*			*GAD-7*			*PHQ-9*			*GAD-7*		
	*OR*	*95% CI*		*OR*	*95% CI*		*OR*	*95% CI*		*OR*	*95% CI*	
*Education*												
No education	0.97	0.26	3.56	0.19	0.04	1.03	2.21	0.41	12.04	0.13	0.02	0.78
Primary education	1.02	0.41	2.58	0.36	0.15	0.83	2.19	0.73	6.60	0.27	0.09	0.80
Secondary education	1.98	0.82	4.75	0.45	0.19	1.02	3.20	1.19	8.62	0.41	0.16	1.04
Higher education (reference)	1.00			1.00			1.00			1.00		
*Household wealth*												
Poorest (reference)	1.00			1.00			1.00			1.00		
Poorer	0.67	0.25	1.79	1.12	0.42	2.97	0.67	0.25	1.80	1.11	0.40	3.06
Middle	1.28	0.44	3.71	1.02	0.40	2.64	1.09	0.27	4.46	0.75	0.28	2.04
Richer	1.28	0.59	2.77	1.66	0.74	3.72	0.64	0.21	2.00	0.79	0.30	2.09
Richest	2.09	1.04	4.20	1.41	0.63	3.19	0.86	0.25	2.90	0.43	0.15	1.21
*Employment status*												
Unemployed (reference)	1.00			1.00			1.00			1.00		
Employed	0.57	0.36	0.90	1.17	0.69	1.99	0.51	0.30	0.85	1.06	0.57	2.00
*Age*												
15–19 (reference)	1.00			1.00			1.00			1.00		
20–24	0.55	0.20	1.49	0.83	0.37	1.86	0.75	0.28	2.03	0.87	0.37	2.06
25–29	1.21	0.68	2.16	0.73	0.31	1.67	2.21	0.86	5.64	1.40	0.50	3.92
30–34	1.06	0.59	1.89	1.06	0.49	2.32	1.91	0.70	5.22	1.37	0.41	4.62
35–39	1.68	0.72	3.95	1.02	0.43	2.43	3.39	1.20	9.55	3.25	0.82	12.96
40–44	1.35	0.65	2.81	2.12	0.93	4.88	2.73	0.71	10.45	1.58	0.39	6.46
45–49	1.12	0.48	2.64	1.04	0.36	3.06	2.34	0.58	9.50	1.69	0.48	5.98
50–54	1.36	0.58	3.15	1.72	0.58	5.10	3.12	0.78	12.47	3.03	0.86	10.67
*Place of residence*												
Urban (reference)	1.00			1.00			1.00			1.00		
Rural	0.37	0.22	0.63	0.56	0.35	0.89	0.40	0.22	0.74	0.57	0.31	1.04
*Religion*												
Muslim (reference)	1.00			1.00			1.00			1.00		
Catholic	1.83	0.98	3.44	0.69	0.32	1.49	1.97	0.94	4.11	0.65	0.31	1.39
Zion Christian	0.91	0.43	1.91	0.42	0.18	0.97	0.85	0.39	1.86	0.43	0.18	1.00
Evangelical/Pentecostal	1.05	0.54	2.06	1.06	0.55	2.03	0.94	0.45	1.96	1.03	0.52	2.03
No religion	1.10	0.54	2.21	0.98	0.47	2.03	1.09	0.52	2.27	1.02	0.47	2.21
Other	1.34	0.42	4.33	0.33	0.04	2.59	1.54	0.47	5.03	0.35	0.04	2.80
*Marital status*												
Formerly married (reference)	1.00			1.00			1.00			1.00		
Never married	0.32	0.17	0.59	0.31	0.15	0.64	0.48	0.19	1.21	0.50	0.18	1.40
Currently married	0.28	0.15	0.53	0.34	0.17	0.68	0.33	0.18	0.63	0.34	0.16	0.72

## Discussion

Our findings revealed high levels of symptoms of depression and anxiety among women, that is, 10% and 11%, respectively. Among men, however, only 2% reported symptoms of both probable depression and anxiety. Our observed prevalence rates are lower than reported in some geographical contexts in Mozambique, including the provinces of Sofala and Manica, where Halsted and colleagues [6] found a 19% prevalence rate for depression among the population. It is possible that the initiatives on mental health by the government of Mozambique may be yielding results. For instance, Pires et al. [8] have discussed and commended the good mental health policy program of the government of Mozambique. Similarly, Halsted et al. [6] noted the success of the mental health program in Mozambique, where in the Sofala and Manica Provinces, an estimated 90% of those who sought treatment for any form of mental illnesses at healthcare facilities indicated that they were treated for their condition. Our finding is also supported by the literature as more women than men are observed to have symptoms of depression and anxiety. Unpacking this dynamic, scholars have explained the sex and gender differences to emanate from differences in hormones, the socialization process and life-stress events [9]. Again, Farhane-Medina et al. [20] suggested that while masculinity is a protective factor, femineity may be a risk exposure to anxiety disorders.

Our findings also established that socioeconomic factors were associated with symptoms of depression and anxiety among women and men. For socioeconomic factors, we found that while among women, educational attainment was not significantly associated with depression and anxiety, this was the case for men. Compared to men with higher education, those with secondary education had a higher probability of reporting depressive symptoms. This is consistent with the literature that discusses the protective effect of higher education on the risk of developing depression [21,22]. In contrast, we found that men with no education and with primary levels of education relative to those with higher education were less likely to have symptoms of anxiety. This finding conflicts with that of earlier studies that have established that higher education is negatively associated with anxiety [23,24]. However, in a study focused on young adults in Sweden, it was found that while the level of education was negatively associated with co-occurring anxiety and depression, and depression alone, it was positively associated with symptoms of anxiety alone [25]. Although our study affirms the latter, future studies should explore the association between educational attainment and reporting symptoms of anxiety in Mozambique.

We further established that although among men, household wealth was not associated with symptoms of depression and anxiety, this was not the case for women. Specifically, compared to women in the poorest households, those in the poor, middle, rich and richest households were less prone to indicate they had depression and anxiety. These findings are broadly consistent with the literature, which posits that household wealth tends to protect members from reporting anxiety. For example, in a large European cohort study, it emerged that people coming from households with higher incomes possessed a lesser risk of genetic dispossession to depression. In addition, such individuals were also protected against symptoms of anxiety relative to their colleagues from poor households [26]. Contextualizing this relationship, Zhang et al. [27] explains that poor household wealth may work to adversely impact people’s health insurance investments and neighbourhood relationships, which may directly lead to feelings of anxiety.

It emerged in our findings that among women, the unemployed were more likely to have symptoms of depression and anxiety, while among men, unemployment was only associated with depression. This finding may not be surprising given that the unemployed may not be well-positioned to meet their financial and economic obligations. This reality often works to heighten their levels of stress and depressive moods. According to Arena et al. [28], an employed status plays a critical role in ensuring people’s survival and satisfying their basic human needs centred around time structure and social connection. These are important and underlie positive mental health and well-being.

We also note that demographic factors were associated with depression and anxiety in Mozambique. Compared to the youngest age cohorts, people in the older age categories were more likely to report both depression and anxiety among women, while among men, this was only the case for depression. This finding may contrast with an earlier study by Muanido et al. [5] who noted that in primary health care settings of the Sofala Province of Mozambique, major depressive disorder was negatively associated with age. This means older people were less likely to report depression. Similarly, it was revealed in the United States during the COVID-19 that the prevalence of reporting depression and anxiety decreased with age, with older people having the lowest reported rates of depression and anxiety [29]. However, other authors have also argued that increasing age is associated with life events and chronic conditions that serve as sources of exposure to the risk of depression and anxiety [30,31]. This explanation may also be supported by the findings of the WHO, suggesting people in older age may have heightened levels of depression and anxiety relative to younger people [1].

Furthermore, among women, those with non-Muslim religious beliefs as well as those identifying with no religion were less likely to have symptoms of depression and anxiety compared to their Muslim counterparts. While some studies have pointed to the association of holding religious beliefs with depression and anxiety symptoms [12], others posit that Christian religious affiliation may be associated with a relatively better coping mechanism, a dynamic that may be underlying our findings [13,32]. We further noted in our findings that while the currently married women were less likely to report depression compared to formerly married women, for men, the currently married and never married were less likely to have depression compared to their formerly married counterparts. For anxiety, the currently and never-married women and the currently married men had a lower probability of reporting symptoms. This may be explained by Halsted et al. [6] who posit that marriage unions may provide social support, which is observed to be beneficial for building resilience against depression, anxiety and other mental health conditions. This finding is also consistent with earlier studies, such as Andrade et al. [33] and Lee et al. [10] who have discussed the impact of being out of a marriage union on depression and anxiety.

We also observed the association between locational characteristics and symptoms of depression among men. Men resident in rural areas had a lower likelihood of reporting symptoms of depression compared to their urban counterparts. This finding contrasts with that of earlier studies, such as Onuh et al. [17], arguing that urban areas tend to have more resources for addressing mental health-related conditions, including depression. In Central Mozambique, Halsted et al. [6] also suggested that being a rural resident was associated with a higher risk of self-reporting depressive symptoms relative to those in urban areas. While this finding calls for further research, Halsted et al. [6] also indicated in their study that rural residents in the country were more likely to seek help for mental health-related conditions than their urban counterparts. Therefore, it is plausible that this may have worked overtime to reduce rural residents’ exposure to depression relative to their urban colleagues.

We make some noteworthy limitations of our study. First, the MDHS’s cross-sectional nature limits our study findings to statistical association and must, therefore, be interpreted with caution. Second, the measure of probable depression and anxiety symptoms is self-reported, which makes it susceptible to response bias from participants. Third, although guided by existing studies and the literature, our study may not have included all the possible factors that may be associated with depression and anxiety in the context of Mozambique. Despite these limitations, our study is among the first to assess at the national level the prevalence and correlates of depression and anxiety among women and men in Mozambique and make policy-relevant recommendations.

While the prevalence rates of depression and anxiety among the general population in Mozambique may be higher than the global average, the country may still be making progress in addressing these mental health conditions. It is instructive for the country to revise or develop a new mental health plan to help sustain the gains made by *Programa de saúde mental 2006–2015*. In this new strategy, it is important to pay critical attention to and specifically target Mozambicans with low levels of educational attainment and household wealth. This can be achieved through sensitization and outreach programs that simplify messages on the need for women and men to undertake screening for depression and anxiety. These outreach programs could also be tailored to teaching these populations about ways to practice self-care, reduce substance abuse and interacting with mental health help centers which can position them to reduce their exposure to depression and anxiety. Additionally, there is need for increased targeting for older age cohorts, the formerly married and men resident in urban areas. Religious bodies or denominations, community-based organization and mass media platforms could be useful partners in reaching these groups with adequate information that would work to strengthen their mental health and reduce their risk of depression and anxiety in Mozambique and elsewhere in SSA with similar context.
